# Micromechanical Force Measurement of Clotted Blood Particle Cohesion: Understanding Thromboembolic Aggregation Mechanisms

**DOI:** 10.1007/s13239-022-00618-2

**Published:** 2022-04-13

**Authors:** Angus J. McKenzie, Barry J. Doyle, Zachary M. Aman

**Affiliations:** 1 Department of Chemical Engineering, The Centre for Long Subsea Tiebacks, Fluid Science and Resources Cluster, The University of Western Australia, 35 Stirling Highway, Crawley, WA 6009 Australia; 2 Vascular Engineering Laboratory, Harry Perkins Institute of Medical Research, Nedlands, and Centre for Medical Research, The University of Western Australia, Crawley, PER Australia; 3 Australian Research Council Centre for Personalised Therapeutics Technologies, Parkville, Australia; 4 BHF Centre for Cardiovascular Science, The University of Edinburgh, Edinburgh, UK

**Keywords:** Embolism, Clotted blood cohesion, Surface-active, Micromechanical force, Particle aggregation

## Abstract

**Purpose:**

Arterial shear forces may promote the embolization of clotted blood from the surface of thrombi, displacing particles that may occlude vasculature, with increased risk of physiological complications and mortality. Thromboemboli may also collide in vivo to form metastable aggregates that increase vessel occlusion likelihood.

**Methods:**

A micromechanical force (MMF) apparatus was modified for aqueous applications to study clot-liquid interfacial phenomena between clotted porcine blood particles suspended in modified continuous phases. The MMF measurement is based on visual observation of particle-particle separation, where Hooke’s Law is applied to calculate separation force. This technique has previously been deployed to study solid–fluid interfacial phenomena in oil and gas pipelines, providing fundamental insight to cohesive and adhesive properties between solids in multiphase flow systems.

**Results:**

This manuscript introduces distributed inter-particle separation force properties as a function of governing physio-chemical parameters; pre-load (contact) force, contact time, and bulk phase chemical modification. In each experimental campaign, the hysteresis and distributed force properties were analysed, to derive insight as to the governing mechanism of cohesion between particles. Porcine serum, porcine albumin and pharmaceutical agents (alteplase, tranexamic acid and hydrolysed aspirin) reduced the measurement by an order of magnitude from the baseline measurement—the apparatus provides a platform to study how surface-active chemistries impact the solid–fluid interface.

**Conclusion:**

These results provide new insight to potential mechanisms of macroscopic thromboembolic aggregation via particles cohering in the vascular system—data that can be directly applied to computational simulations to predict particle fate, better informing the mechanistic developments of embolic occlusion.

**Supplementary Information:**

The online version contains supplementary material available at 10.1007/s13239-022-00618-2.

## Introduction

Arterial occlusion is a common underlying mechanism for ischaemic stroke^22^ and myocardial infarction.^26^ An estimated 795 000 people are subject annually to the incidence of stroke in the United States; 87% of cases are ischaemic, and approximately 79% of cases involve thrombotic occlusion of precerebral and cerebral arteries.^60,78^ The occurrence of cardioembolic stroke has been estimated to account for 20–32% of all ischaemic stroke cases.^9,73^ Atrial fibrillation is the most prevalent cause of coronary artery embolism contributing to acute myocardial infarction.^46,70^ A clinical study revealed that among 407 patients, 40% of stroke cases were primarily dictated by underlying mechanisms of thromboembolism.^15^ The sources of embolic stroke have been evaluated clinically, with primary contributions; aortic atherosclerotic plaque rupture (14–21%), carotid artery disease (10–13%) and atrial fibrillation (18–30%).^8,41,48,60^ Although the source of thromboembolism may be determined, in the development of a blockage, the underlying mechanical interactions between colliding thromboemboli in the arterial tree are poorly understood.

During the build-up phase of thrombus formation, emboli may be shed by action of shear flow^47,80^ around the thrombus due to heterogeneities^25,77^ and instabilities within the structure, while thrombi progressively contract and stabilize.^20^ Cosemans *et al*.^20^ integrated developing hypotheses relating to thrombus growth and stabilization, presenting a series of conceptual mechanisms of the adhesion and subsequent embolization of fibrin-anchored platelet aggregates. Observations taken from *in vivo* and *in vitro* experimental studies involving intentionally damaged arteries in mice and artificial flow chambers, respectively, contributed to these updated concepts.^20^ The mechanisms and systematic developments of thrombo-embolization are often unclear in both *in vivo* and *in vitro* scenarios—deconvolution of physical and chemical contributions is challenging. Chemical mechanobiological heterogeneities and/or deficiencies in thrombotic structures are hypothesized to impart structural vulnerabilities.^18,20,49,67,68^ Additionally, shear stress gradients and rheological disturbances in close proximity to thrombi expose interfaces to higher forces which may promote which may promote embolization.^18,38^ Rhythmic pulsatile flow may further exacerbate these phenomena, although this is poorly understood.^20^ Fibrin was found to resist shear-induced embolization of growing thrombi by 12–28 fold, emphasizing the importance that fibrin plays in thrombus stabilization, resisting compromises in structural integrity.^18^ Simulations have revealed that clot permeability also plays a critical role in mechanically-induced embolization of material from the surface of thrombi, particularly when coupled with elevated shear rates (1000–2000 s^−1^) that provide continual microembolization.^80^

Dynamic Doppler ultrasound detection is a non-invasive technique that has revealed physical characteristics of emboli, as well as being a potential marker for stroke risk, in clinical and experimental environments.^52–54,69^ However, limited information is known about the interplay of numerous emboli in the development of a blockage, as there is a lack of experimental data which can be utilized as a input to computational fluid dynamics models for tracking embolic particle fate.^29^ Simulating emboli trajectory to numerically predict embolic fate, through complex geometries such as arterial bifurcations,^1,17,62^ has been of interest in the literature. Embolic particle transport has been computationally modelled within reconstructed three-dimensional human anatomical models of arterial sections; aortic arches, circle of Willis, bifurcations, carotid and vertebral arteries.^16,24,61–63^ Embolus and vessel wall anatomical and biomechanical properties were studied. Mukherjee *et al*.^62^ outlined a momentum balance governing fluid-particle interactions for emboli, where the motion of particles within simulated vasculature is modelled via a modified form of the Maxey–Riley^55^ equation. Fabbri *et al*.^24^ detailed a fundamental collision principle involving a restitution coefficient, computing the momentum exchange for particle collisions. For simplicity, this coefficient was set to 1.0, implying perfectly elastic collisions—a value of zero would imply particle-particle coherence. Mukherjee *et al*.^62^ acknowledged that improved numerical solutions may require knowledge and implementation of the dictating parameters relevant to contact force between emboli (and between emboli and the vessel wall), to better assess embolic fate in the development of arterial occlusions.

**Figure 1:**
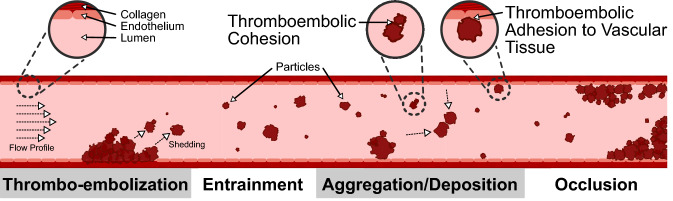
Conceptual four-stage picture of the major developments of thrombo-embolization through to vessel occlusion. The first stage of this four-stage conceptual mechanism is adapted from Cosemans *et al*.^20^ The multi-stage nature of this concept is in the style of Turner (in collaboration with J. Abrahamson^75^)

As an extension to shear-induced embolization phenomena conceptualized by Cosemans *et al*.,^20^ a four-stage conceptual mechanism to describe thromboembolic-induced occlusion is introduced pictorially in Fig. 1. Downstream of *thrombo-embolization* sites, particles are pictured as being *entrained* within blood flow, before colliding with neighbouring particles and/or the vessel wall(s). Thromboembolic *aggregation/deposition* phenomena may precede the onset of full-scale vessel *occlusion*, whereby cohesive particle-particle collisions enable the formation of larger stable aggregates, and adhesive particle-wall collisions result in particle deposition. Along with complex mechanobiology that may dictate embolic-aggregation mechanics,^42^ these phenomena may be critical in establishing thrombo-embolic occlusions in non-stenotic sections of the arterial tree.

The primary focus of this manuscript is to provide a pathway to understand both the contact mechanisms that may be at play between thrombo-emboli *in vivo*, and the degree that clot cohesion is sensitive to the chemical environment. This is inclusive of arterial (‘white’—platelet-rich) and venous (‘red’—erythrocyte-rich) thrombi. In this work, we have adopted an experimental strategy to quantify the inter-particle cohesive force between model clotted blood particles of porcine origin in a static *in vitro* experimental system with a micromechanical force (MMF) apparatus—the inaugural cardiovascular application.

MMF technology was originally developed to visually observe solid–fluid interfacial phenomena that occur in subsea oil and gas pipelines.^72^ The technique has previously been used to study cohesion between microscopic solids in modified oil phases that may block pipelines by forming larger stable aggregates; clathrate hydrate, asphaltenic particles.^58^ Additionally, adhesive properties (solid–wall interactions) have been investigated with this technique,^4,6^ and thromboembolic adhesion (in light of deposition phenomena) is an area of proposed investigation in this space. MMF measurements have been utilized in slurry-viscosity models to predict the occurrence of pipeline blockages.^13^ Surface-active injection chemicals have been found to impact the measurement by reducing solid–fluid interfacial tension.^7^ The results in this manuscript may better inform the mechanisms of thromboembolic cohesion and the possibility of larger aggregate formation, posing higher occlusion risk in the arterial tree—an area that has not been previously reported upon in the literature. Contributions of mechanistic understanding supplement the computational models reported in the literature, and provide a platform to more representatively simulate embolic fate and occlusion development.

## Methods and Materials

### Micromechanical Force (MMF) Apparatus

**Figure 2:**
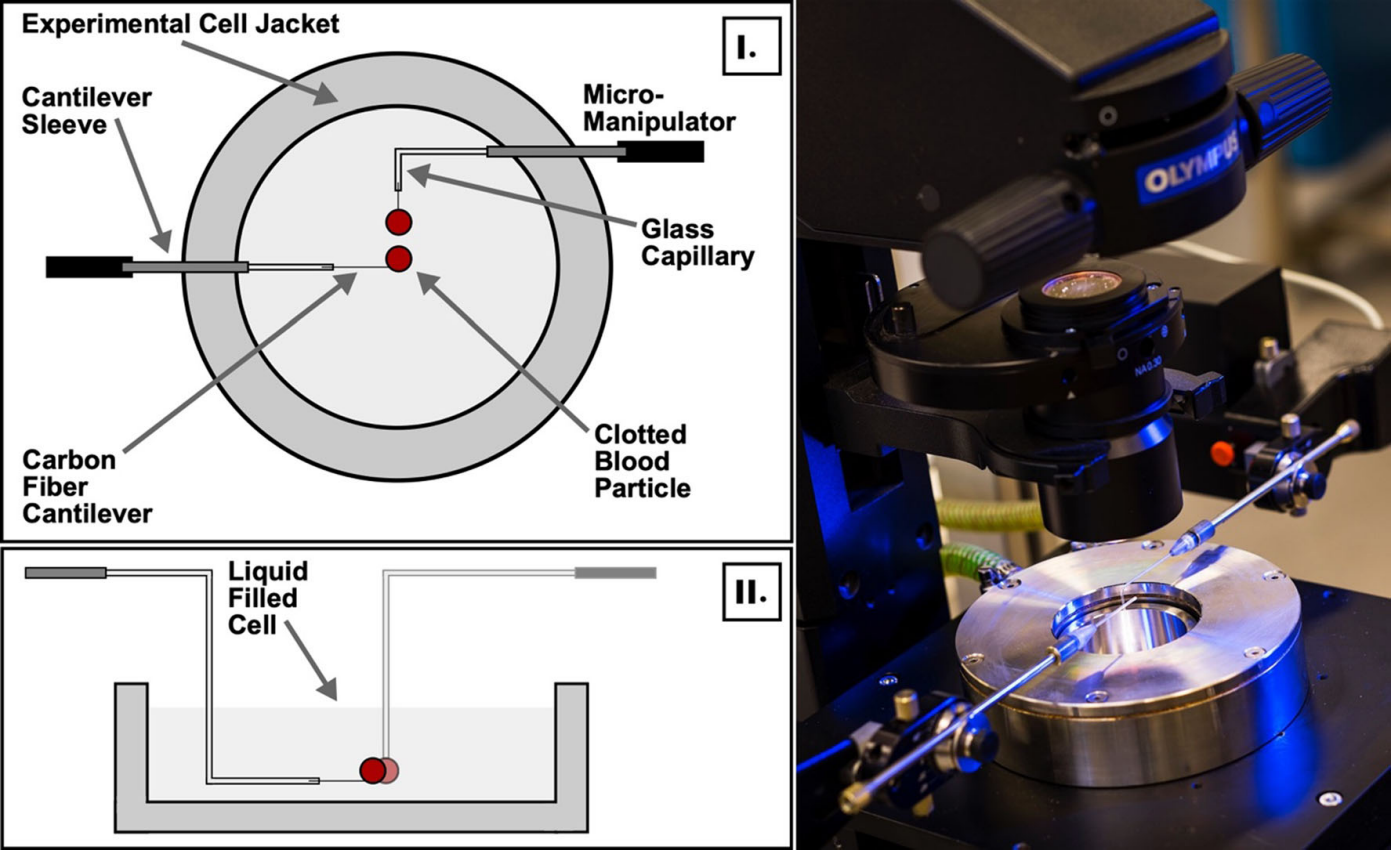
Schematic of experimental system—overhead view (I) and cross-sectional view of cantilevers and attachments (II) The micromechanical force apparatus consists of an inverted light microscope and experimental cell resting on an active pneumatic isolation table (right).

A third-generation MMF apparatus was applied to observe and measure clotted blood inter-particle separation force. The same force measurement technique applied by Morrissy *et al*.^59^ was utilized in this work, which was adapted from previously reported methods^4,5^—the reader is referred to these for further detail. The apparatus consists of an Olympus IX-53 inverted light microscope (Fig. 2) fitted with digital recording equipment, with images captured and analysed via Olympus cellSens software.

**Figure 3:**
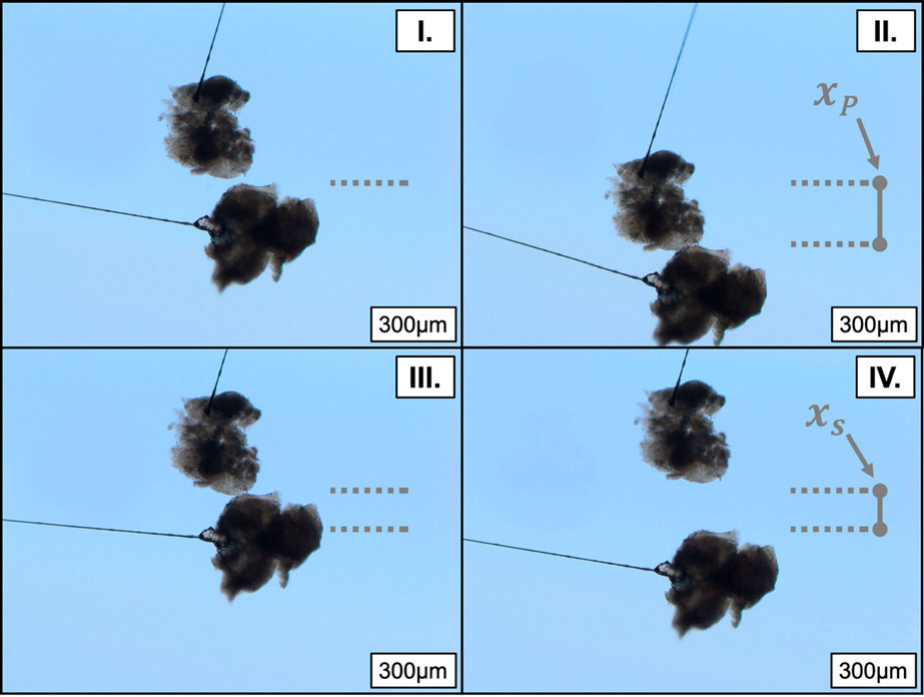
A four-step pull-off procedure allows for direct calculation of the preload and separation force as the product of the spring constant (*k*_spring_) and preload or separation displacements (*x*_P_, *x*_S_), respectively. The blue background is an optical filter given by the microscope to help distinguish the particle-continuous phase interface.

An experimental cell with a glass transparent viewing window was placed atop the microscope stage, where it was filled with a liquid phase to suspend particles and held at ambient temperature conditions (22–24C). The microscope and cell assembly were placed atop an active pneumatic vibration isolation table (Kinetic Systems 9100 Series Vibration Control Workstation) to minimize the effect of external forces on particle positioning. The cell contained two glass capillaries (1000 μm internal diameter) that were held by stainless steel arms (Olympus) and secured in micro manipulators with sub-micron precision (Eppendorf & Narishige Group). A 7 μm external diameter carbon fibre filament (Fibre Glast Developments Corporation) was secured within each capillary tube by epoxy adhesive—this procedure does not impact global properties of solids, e.g. elasticity, morphology etc.^58^ Epoxy was not located close to the inter-particle contact area. A schematic of the experimental cell from an overhead view, along with cantilevers suspending clotted blood particles is presented in Fig. 2. Additionally, a cross-sectional representation of the stationary cantilever and attachments is presented here, where a liquid level in the experimental cell is demarcated by a solid light grey area. The separation force between solid particles was measured indirectly using a four-step method^81^ (pictured in Fig. 3): (I.) the top particle was manipulated into contact with the bottom particle, providing a preload force; (II.) the particles remained in contact for a period of time, reaching mechanical equilibrium; (III.) the top particle was then vertically raised (with respect to Fig. 3) at constant velocity until the particles separated; and (IV.) the displacement of separation was captured visually via recording.

Hooke’s law (Eq. (1)) is applied to calculate the force of separation, which is the product of the spring constant, ${k}_{spring}$, and the distance of separation, ${x}_{s}$ [*µ*m].

1 $$F={k}_{\rm{spring}}.{x}_{s},$$

The spring constant, ${k}_{\rm{spring}}$, of the carbon-fiber cantilever was calculated from the geometric and material properties of the fiber using Eq. (2):

2 $${k}_{\rm{spring}}=\frac{3\pi E{d}^{4}}{64{L}^{3}},$$

where, *E* is the elastic modulus of the material, *d* is the diameter of the fiber, and *L* is the length of the fiber. The elastic modulus, fiber diameter, and fiber length had relative uncertainties: 6%, 7%, and 0.1%, respectively. The elastic modulus of the carbon fiber was previously reported as 250 GPa^35^. Fiber lengths were typically between 3000 and 4000 *µ*m. Morrissy *et al*.^58^ benchmarked values of fiber spring constants determined from Eq. (2) against that of a reference tungsten wire, and found consistency within the experimental uncertainty—see supplementary materials for further information. The spring constant of the carbon fiber was used to determine the force at cohesive failure from the measured lateral displacement. To compare the results for particles of different sizes, the measured force was divided by the harmonic mean radius of the particle pair (R^*^ in Eq. (3)).

3 $${R}^{*}=\frac{2{R}_{1}{R}_{2}}{{R}_{1}+{R}_{2}}.$$

These measurements required the implementation of fresh particle pairs for each experiment, a set of blank cohesion measurements was conducted to obtain a ‘baseline’ (control) measurement for each system. For each experimental data point (excluding the contact time study), twenty pull-off trials were performed to obtain a statistically-representative sample. The uncertainty bounds represent a 95% CI.

### Materials: Porcine Blood Samples

Pigs and humans exhibit similar anatomical dimensions and characteristics of the cardiovascular system.^43^ Porcine blood clots were selected as model solids for this study for which the cohesive properties were investigated.

#### Porcine Blood Handling and Storage

Porcine blood was sourced from the Linley Valley Pork Processing Facility, Western Australia; all work was carried out under institution ethics approval. Blood clots in un-coagulated blood were isolated, transferred to and frozen in containers. The material within these samples was defrosted when required, and stored within a refrigerator at 4°C for one week, before being disposed. No chemical treatment/preservation of samples (i.e. no anticoagulation) was carried out, to eliminate the possibility of modifying samples with introduced chemicals, chemically and morphologically—the MMF measurement is sensitive to the chemical environment of the continuous phase.

#### Porcine Clotted Blood Particle Preparation

Before each experiment, a sample of clotted blood was rinsed in DI water, to minimize the transfer of blood to the experimental cell (static liquid phase), and the leaching of intra-clot liquid blood components. Clotted blood was rinsed in DI water, freeing clots of native liquid blood components. Preliminary work suggested that non-rinsed clots leached liquid blood that impacted the reproducibility of the measurement. Clotted particles on the order of 300–900 *µ*m were isolated with a scalpel under microscope—see the supplementary materials for further details. Particles were partially air-dried before epoxied to tips of carbon-fibre cantilevers, before being transferred to the experimental space. Particles were left suspended in the continuous phase for thirty minutes to stabilize, before any mechanical manipulation/contacting was applied.

#### Chemical Modifications to the Continuous Phase

Alteplase, tranexamic acid, and aspirin are chemistries that are introduced to the bloodstream in the occurrence or prevention of conditions such as acute ischaemic stroke, haemorrhage, and cardiovascular inflammation, respectively.^19,34^ The MMF measurement is sensitive to particular chemical environments, whereby introduced surface-active chemistries may adsorb to the solid–liquid interface and modify the interfacial tension across the contact area^7^—see supplementary material for details. An objective of this work was to assess the activity of these chemistries, by adsorption or other complex chemical interactions, at the clot-liquid interface, whereby the impact on the MMF measurement was investigated. These agents were introduced to the experimental space, and tested to a higher-end limit in additive mass fraction—dictated by cell liquid volume or solubility constraints. Stock solutions of additives (all additives tested) were solubilized in DI water [wt%], respectively, and diluted gravimetrically to measure the inter-particle separation force as a function of additive mass fraction. These solutions were injected independently into the continuous phase in the absence of any other chemical additives.

In a separate experimental campaign, naturally-derived surface-active components of blood were independently introduced to the liquid phase, assessing their impact on the measurement. Slaughterhouse blood contains useful bioactive components, such as proteins, summarizing select properties of theses constituents; albumin, fibrinogen, haemoglobin, *α*-globulins, *β*-globulins and *γ*-globulins.^10^ There is verification in the literature that proteins, which are commonly amphiphatic macromolecules, readily adsorb and accumulate at high-energy solid–fluid interfaces.^30,64^ Protein fractions make up 6–8 wt% of plasma, where in pigs, albumin constitutes the dominant proportion of plasma proteins.^10,27^ Unmodified porcine serum (a combination of protein fractions) and a hydrolysed fraction of albumin proteins (single fraction), were tested independently to assess their impact on the measurement.

Frozen Gibco® Porcine serum of New Zealand origin (ThermoFisher Scientific), subject to sterile-filtered treatment, was stored in a laboratory freezer at – 10°°C, defrosted when required. A serum protein electrophoresis was performed to determine the protein component fraction in a non-haemolysed sample. This was conducted externally by VetPath Laboratory Services (Perth, Australia). The densitometer trace of the electrophoresis performed independently for this work evaluated these protein component fraction as; 48 wt% albumin, 2.9 wt% ${\alpha }_{1}$-globulins, 14.4 wt% ${\alpha }_{2}$-globulins, 5.4 wt% ${\beta }_{1}$-globulins, 9.7 wt% ${\beta }_{2}$-globulins, 19.6 wt% $\gamma$-globulins. As haemoglobin is contained within the cellular fraction, and fibrinogens are removed during the production of serum, albumins and the *α*-globulins, *β*-globulins and *γ*-globulins made up the remaining fraction of proteins within the serum sample. The total protein content in the serum sample was 8.3 wt%, and the respective albumin content was 3.98 wt%, which is in good agreement with published work.^10^ A lyophilized powder (agarose gel electrophoresis) of albumin from porcine serum (Sigma Aldrich ≥ 98%) was hydrolysed in DI water on the order of 1 wt%–the most dominant protein fraction by mass in the densitometer trace.

10 mg of Actilyse*®*, manufactured by Boehringer Ingelheim, was reconstituted in 10 mL of DI water using a transfer cannula. Alteplase is a glycoprotein recombinant tissue plasminogen activator (rt-PA) that promotes the conversion of plasminogen to plasmin in the process of fibrinolysis^76^—critical in maintaining haemostatic control. It is a fibrin-specific agent, whereby it interacts with fibrin-fluid interfaces that bind plasminogen, producing plasmin that acts to dissolve fibrin-rich thrombi by cleaving fibrin strands.^57^

Tranexamic acid [trans-4-(aminomethyl)cyclohexanecarboxylic acid] of 99+% purity from Glentham Life Sciences was solubilized to 4.97 wt % in DI water. Tranexamic acid is a antifibrinolytic agent that is administered to patients who are experiencing or susceptible to haemorrhage.^23^ *In vivo*, the agent acts to retard fibrinolysis by block lysine binding sites on plasminogen.^32^

Aspirin (99+% acetylsalicylic acid) from Sigma-Aldrich was hydrolysed in DI water to 0.1 wt%, using a magnetic stirrer at ambient temperature conditions, approaching a super-saturation condition. No thermal stimulation was induced in the dissolution process to enhance the solubility. Acetylsalicylic acid hydrolysis forms salicylic and acetic acid.^39^ Aspirin has historically been used to prevent cardiovascular disease, as an effective suppressant of platelet aggregation.^37^

#### Baseline Measurement Acts as Reference

As a primary objective, establishing a statistically repeatable base system was critical to understand which measurable physio-chemical parameters impact the measurement. Operating heuristics/conditions for the physio-chemistry of the experimental system were established based on the data presented in the results & supplementary sections. The physico-chemical parameters that may govern the measurement are listed in the supplementary information section.

#### Selection of a Baseline System

A model continuous phase of DI water was selected to establish the ‘baseline’ (control) measurement—previous work indicated that targeting a chemically simple system to investigate the effect of mechanical-related parameters (e.g. pre-load force, contact time) is critical. As blood is an aqueous suspension of cells, proteins, electrolytes etc., utilising DI water as the continuous phase is the simplest representation of its vascular counterpart. Given the mechanical nature of the cohesive force mechanism, the distribution of the measurement may be induced systematically for each pair of particles with successive inter-particle contacts, where the confidence in the measurement should be represented via a statistical approach. To eliminate any systematic offset and maintain reproducibility, a maximum of twenty contacts for each particle pair was performed. Over extended contact quantities, it was observed that applied forces deformed contact locations at the interface, whereby the data analysis outlined clear systematic offsets in the measurement—induced deformation reduced the cohesive measurement. Over extended contact repetition, we hypothesize that the interfacial contact area is reduced by decreased surface roughness via particle deterioration, which may be a critical governing parameter for the measurement. Additional measures were introduced to reduce artificial impact; particles were contacted at different locations for each measurement, and rested for ten seconds between pull-off measurements. Non-repeating contact locations may have exposed particles to variable contact areas, thereby more representatively targeting the deterministic nature of cohesion for the material in DI water.

### Baseline Study: Clotted Blood Particle Cohesive Force as a Function of Contact/Pre-load Force

The range of preload force that could be studied was determined based on the available microscope field of view—the preload force is normalized based on the harmonic mean radius of the particles and the length of the stationary cantilever. The pre-load force [*µ*N] was applied by displacing the stationary particle with the moving particle with a pre-determined pre-load displacement ${x}_{\rm{P}}$, as described in Eq. (4). This is shown pictorially in Fig. 3. A standard contact force (${F}_{\rm{CF}}$) of 1.7 [mN/m] was applied.^3^

4 $$ x_{{\text{P}}} [\mu m] = \frac{{F_{{{\text{CF}}}} \left[ {\frac{{mN}}{m}} \right]\times R^{*} \left[ m \right]\times\left( {\frac{{1000}}{1}} \right)\left[ {\frac{{\mu m}}{{mm}}} \right]}}{{k\left[ {\frac{N}{m}} \right]}}. $$

**Figure 4:**
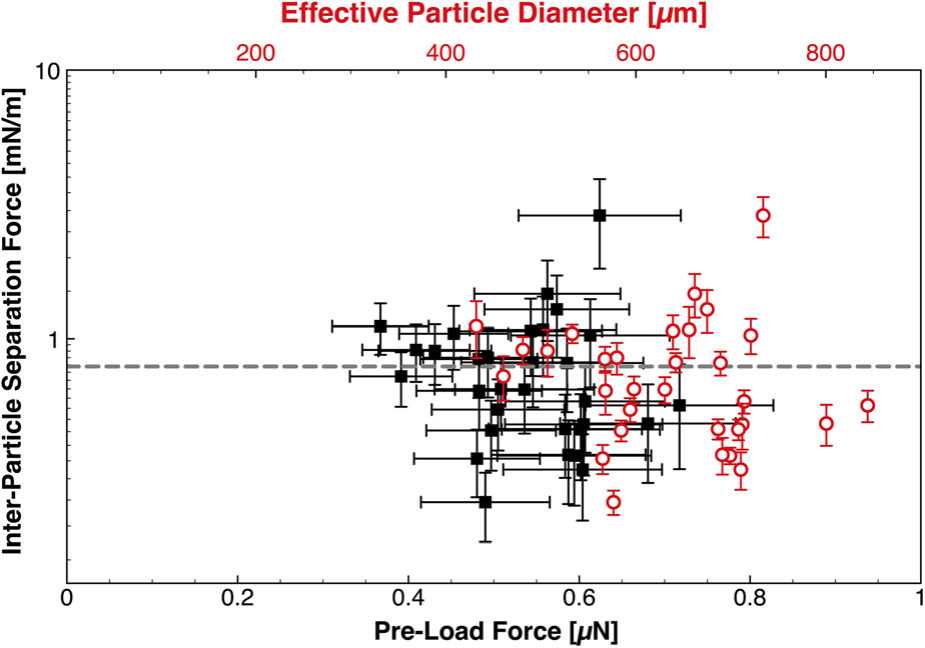
Inter-particle separation force as a function of pre-load force (and effective particle diameter), where the error bounds represent a 95% CI. A dashed grey line demarcates the mean baseline inter-particle separation force.

These measurements were performed at low contact time (10 s each). Each data point in Fig. 4 represents a mean of twenty measurements for a unique particle pair with an associated 95% CI for both the separation and preload forces, respectively.

#### Baseline Study: Clotted Blood Particle Cohesive Force as a Function of Contact Time

A unique particle pair was used for each experiment—the same two particles were not tested at dissimilar contact times. Observations of increased mechanically-induced deformation at the point of contact were made as the contact time between particles increased. To eliminate any systematic drift in the measurement, less individual measurements were conducted per particle pair for extended contact times. The amount of total individual measurements collected per data point is listed in Table 1 with an associated number of measurements conducted per particle pair, before being discarded for a new particle pair.

**Table 1:** Number of measurements performed for a range of inter-particle contact times.

Inter-particle contact time	Total individual measurements	Number of measurements conducted per particle pair
10 s	620	20
20 s	50	20
1 min	35	10
2 min	35	10
5 min	40	5
20 min	25	5
1 h	15	5
18 h	4	1

#### Baseline System Distributed Force Properties

Thirty-one data sets were compiled to establish a baseline measurement of inter-particle separation force [mN/m] between clotted blood particles suspended in DI water—a comparable number of data sets to previously established baseline measurements using the MMF technique.^3^ The measurements reported in Section represent a collection of individual measurements extracted from data sets for baseline experiments (low contact time: 10 s) of twenty pull-off trials per particle-pair. The properties of the data were treated statistically via a Shapiro-Wilk to test for lognormality at 95% confidence—the test failed to reject the null hypothesis. The lognormality test was performed for the group properties at the same confidence level—6 of 31 data sets failed to reject the null hypothesis. A lognormal distribution function was fit to the raw data of 620 individual measurements, which is demarcated by a shaded grey area in Section 8.2 (supplementary material). The purpose of hypothesis testing was to assess and visualize the lognormality in one or more variables involved in the measurement basis, as well as quantify an appropriate 95% CI.

#### Testing Involving Modification of the Continuous Phase

The data presented in “Measurement Sensitivity to the Chemical Environment” section of this work represents a mean of two data sets of twenty measurements each, where two unique particle pairs were tested, with a corresponding 95% CI reported.

## Results and Discussion

To understand how the measurement is affect by the chemical environment, the mechanical parameters of the measurement were independently explored (i.e. effect of pre-load force, contact time)—while maintaining control of the continuous phase chemistry. However, due to the chemical heterogeneity and diversity of the clot, this makes functional interrogation of the mechanobiology not possible without chemical modification to the clot. With an established repeatable baseline, the chemistry of the continuous phase was then modified, to assess the sensitivity of the measurement in relation to modification of the chemical environment.

### Baseline System

#### Contact/Pre-load Force Dependence

The clotted blood inter-particle separation force was calculated as a function of preload force (the force applied to a stationary particle during step II. of the four step pull-off technique—see Fig. 3), to derive information on the nature of the dominant cohesion mechanism in Fig. 4.

Israelachvili^36^ described a two-phase solid–solid cohesion model which may be active at length scales of greater than 10µm, where the force is related to the energy bound at the interfaces between the solids, and the solid and surrounding liquid medium (Eq. (5)).

5 $${{F}_{\rm{ad}}=-3\pi R}^{*}{\gamma }_{\rm{SL}}.$$

Here ${F}_{\rm{ad}}$ is the force of adhesion/cohesion, ${R}^{*}$ is the harmonic mean radius of the particles, and ${\gamma }_{\rm{SL}}$ is the interfacial tension between the solid and liquid phases. Johnson, Kendall, and Roberts (JKR) and Hertz^40^ theory describes the deformation of elastic solids with applied force, however these theories do not account for surface roughness. Since solid–solid cohesion depends on the contact area between particles, the force required to separate elastic particles should increase with preload force—as elastic deformation leads to increased contact area. The data in Fig. 4 shows that there is no clear dependence of preload force on the force required to separate the particles, and hence the solid–solid cohesion mechanism described by Israelachvili^36^ is not applicable to describe these clot-liquid interfacial phenomena. Without maturity in understanding of the applicable contact mechanics, application of this data to computational simulation may be more limited, necessitating further study.

#### Contact Time Dependence

Recirculation and/or stagnant zones are low shear environments in the arterial tree, which may be favourable for both embolic deposition and aggregation^31^—inter-particle contacts may be lengthier than in high shear environments. These zones may create situations where particles experience high residence time, and may be induced on the downstream side of a growing thrombus, a flap/tear due to plaque rupture, or a poorly design prosthetic valve/ geometric flow separator.^74,79^ To understand the cohesive nature between thromboembolic particles as a function of contact time, it is critical to assess these mechanisms on a wide-basis that includes a ‘worst case scenario’ (contact times that may be outside the range of physiological significance). Experimental *in vitro* work in well-characterised shear fields has revealed that platelet and fibrin aggregation occurs on the order of at least 10 s residence time.^33,66^ Although these aggregation phenomena occur on the nanoscale (several orders of magnitude smaller length scale than the diameter of thromboemboli), this provided some context for this contact time dependence investigation.

**Figure 5:**
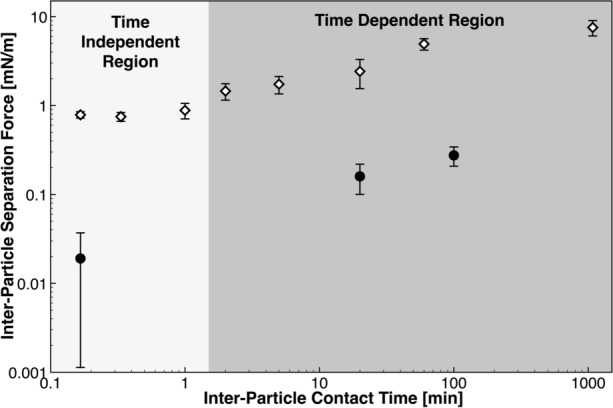
Inter-particle separation force as a function of inter-particle contact time. The open diamond data denote measurements taken in an unmodified continuous phase (DI water). The black circles denote those measurements taken in a continuous phase where 4 wt% whole blood was injected. The error bounds represent a 95% CI in the measurement.

The solid–solid cohesion mechanism, described previously, is time independent and has a purely mechanical outcome. To assess the time dependent nature of the contact mechanics, particles were held in contact for variable periods of time (step II. of the four-step pull-off technique [maximum pre-load force and displacement]—see Fig. 3). Evident in the data presented in Fig. 5, a transition exists where the inter-particle separation force becomes dependent on time after a threshold on the order of approximately one minute. Below this transition, there is no statistical dependence on time; the measurements taken at 10 seconds form the baseline distribution covered in the supplementary material, and is used as a basis for assessing the sensitivity of the measurement on the composition of the chemical environment. Beyond this transition, the magnitude of the measurement deviates from the established baseline (0.79 ± 0.06 [mN/m]) and progressively increases as the contact time (held at the point of) is extended for the range investigated.

The contact mechanics in these two regions may be governed by two distinct mechanisms—over extended time, the contact area between the interfaces may increase substantially due to time-dependent elasticity for the load exerted between the particles and/or a chemical reaction (e.g. growth, sintering, annealing) at the interface may be taking place. Although no growth/sintering (or reaction) was observed between the particles, there may have been activity below the detection limit of the apparatus. In a separate set of experiments, 4 wt% of unmodified whole blood from which clotted blood was taken, was injected and dispersed in the continuous phase. The data in Fig. 5 highlights that at the baseline inter-particle contact time (10 s), the magnitude of the measurement is reduced by an order of magnitude. Note: as depicted by the 95% CI, the lower bound falls below the detectable limit of the apparatus (~ 0.01 mN/m), as no measurable pull-off displacement was observed for some of the individual twenty measurements that form this data point. The technique is limited by visual transparency—it is not possible to measure clot cohesion within a pure sample (i.e. 100 wt%) of whole blood due to opacity. Chemical modification of this continuous phase with surface active material contained within whole blood may explain these results—phenomena that has been previously observed, and example data is listed in the supplementary material.^7^ Unmodified whole blood contains a wide variety of proteins which are surface-active amphiphatic macromolecules that may adsorb and accumulate at high energy solid–fluid interfaces.^10,30,64^ Complementary to the contact time study, inter-particle separation force was also measured for particles held in contact for twenty, and one hundred minutes, respectively. Similarly, with the unmodified continuous phase of pure DI water, a time-dependent increase in the separation force was observed.

The authors of this work declare that this baseline data should be treated with care—it may not represent the order of magnitude of thromboembolic cohesion *in vivo.* The in-vitro environment, particle preparation methods and storage conditions are far from the nature of the arterial lumen in which these interactions may be taking place. This work provides a method to understand the implication of the contact mechanics that may be apparent, while developing an improved understanding of the dominant physio-chemical variables that govern the cohesion mechanics—maturity in this understanding may support the application of inputs to computational particle tracking models.

#### Measurement Sensitivity to the Chemical Environment

Surfactants have previously been characterized as having a unique ability to adsorb to the solid–fluid interface, working as a central mechanism in reducing the tendency for particles cohesion.^7^ A series of common pharmaceutical agents (cardiovascular-active) and natural species extracted from whole blood were tested. These species existing *in vivo* may limit the degree to which thromboemboli can cohere and form larger stable aggregates that have an increased likelihood of causing an occlusion.

The authors of this work acknowledge that to expand on the understanding of thromboembolic cohesive mechanisms that this work presents, characterisation of the clots utilised for these measurements is of significance. However, due to the chemical complexity and structural heterogeneity between samples, an in-depth study would be required and presents an area of possible future work—the complex mechanobiology that may influence the measurement cannot be addressed with this technique: receptor-ligand interactions, or activated-platelet interactions (e.g. von Willebrand factor [vWF]). This would require manipulation of raw ingredients to form particles suitable for this type of measurement procedure. This may inform the degree to which individual thromboembolic constituents impact the measurement—as such, the clotted blood particles utilised in this work can be treated as ‘model’ clots, and a statistically significant data set of ‘baseline measurements’ provides reference for assessing the impact of chemical additives to the cohesive nature between these solids. Further to these challenges associated with studying aspects of clot chemical heterogeneity that may contribute to thromboembolic aggregation *in vivo*: the dependency of thrombus composition on stroke etiology has been found to be unclear, and thromboemboli retrieved from the middle cerebral and intracranial carotid arteries have possessed comparable histology.^50,51,65^ These analyses have indicated that intrinsically, thrombotic material are largely heterogeneous arrangements and commonly composed largely of commingled fibrin-platelet strands, enthrocytes, and nucleated cells (both monocytes and neutrophils).^50,51^ Boeckh-Behrens *et al*.^12^ reported that thrombi obtained via thrombectomy^28^ from thirty-four patients were composed of fibrin (60% ± 21), erythrocytes (32% ± 23), leukocytes (8% ± 5).

Performing these measurements at cardiovascular temperatures would be more representative; based on previous studies, the authors believe that these thermodynamic effects would be active within the resolution of the measurement, and hence was not a focus in establishing this method—there is no third phase present promoting a capillary bridge whose thickness is governed thermodynamically.

#### Blood-Derived Proteins

**Figure 6:**
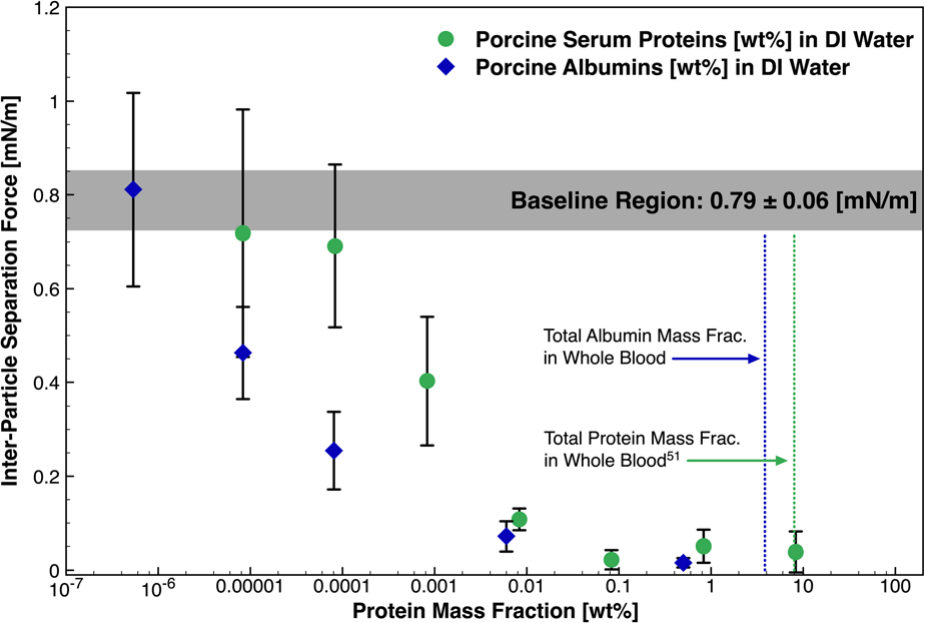
Inter-particle separation force as a function of serum protein and albumin mass fraction in the continuous phase. This data has been treated to account for the total protein content [wt%] in the continuous phase. The error bounds signify a 95% CI in the measurement.

The data in Fig. 6 highlights a reduction in the inter-particle separation force between clotted blood particles, compared to the base system (grey region), in the presence of proteins derived from blood. Note: data involving porcine serum is scaled for the mass fraction of proteins, in order to compare the data sets—i.e. 8.3 wt% porcine serum proteins is equivalent to 100 wt% unmodified serum in the experimental cell. Unmodified serum or albumin presence in the experimental cell did not visually alter particle morphology. At a serum dilution of 1 ppm (0.0001 wt%), the measurement is within the statistical confidence of the baseline. An intermediate region follows where continued adsorption of material to the solid–liquid interface may be evident, however this may not be the only applicable interaction of these species with the clot. At a mass fraction of 1 wt% or higher, the force measurement is reduced by an order of magnitude, when compared to baseline force, and to within the detection limit of the apparatus.

At 0.1 ppm, hydrolysed albumin proteins reduced the cohesive force by 43%. At a respective mass fraction of serum in DI water, the measurement is within the statistical significance for the albumin fraction hydrolysed in DI water for the same order of magnitude dilution. Although some preferential or competitive adsorption may be occurring between the components of the serum proteins at the solid–fluid interface, the data emphasizes that the activity between protein additives are statistically indistinguishable. The data highlights that the force between colliding embolic solids *in vivo* may be lower than the baseline system, providing a method to assess the surface-active nature of blood components.

#### Pharmaceutical Agents

**Figure 7:**
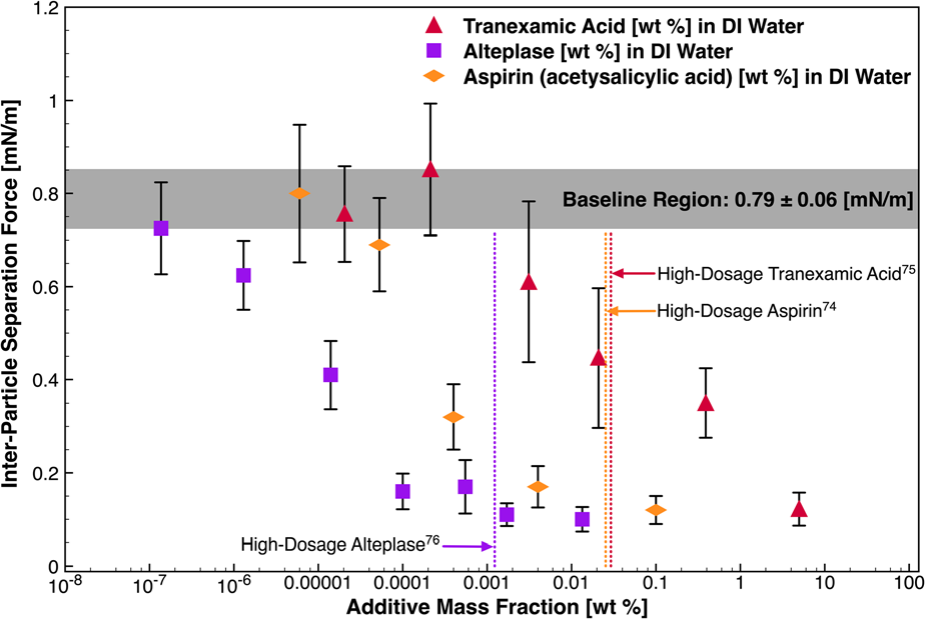
Inter-particle separation force as a function of additive mass fraction in the experimental system. The red triangles, orange flat diamonds and purple squares represent data sets involving a continuous phase of tranexamic acid, aspirin and alteplase, respectively, solubilized in DI water [wt%]. The error bounds signify a 95% CI in the measurement.

Alteplase, tranexamic acid and aspirin were tested, respectively, as these agents are widely used to target various conditions where thromboembolic solids may be present. Dilutions of hydrolysed forms of these chemistries were injected into the experimental cell and did not visually alter clotted particle morphology—a necessary outcome in the MMF procedure. On the order of 1E−05 wt% aspirin diluted in the experimental cell, the introduced chemistry fails to deviate the measurement from the baseline, as highlighted in Fig. 7. An intermediate region follows where adsorption of material may be evident, as the cohesive force decreases with increasing mass fraction. At 4E−04 wt% mass fraction, the force measurement is reduced by 60% from a baseline magnitude. The measurement is reduced by 85% at 0.1 wt%, where beyond this condition, aspirin could not be readily solubilized at ambient conditions. Vertical dashed lines are provided for reference, which represents the in vivo mass fraction for high-end alteplase (0.9 mg/kg), tranexamic acid (1500 mg) and aspirin (1300 mg) dosages, respectively, for an average sized adult.^14,45,56^

Alteplase adsorbs and is active at an order of magnitude lower than aspirin—paralleling the performance and demand for the drug in treating myocardial infarction in the clinical environment.^2^ We hypothesize this behaviour to be a direct result of surfactant adsorption to the clot-liquid interface. As indicated by the vertical dashes lines, which represent the mass fraction of these chemistries in the blood stream at standard clinical (higher-end) dosages, depreciable reduction in the measurement is evident at these conditions for alteplase and aspirin. There is a strong correlation between clotted blood particle cohesive force reduction, and the mitigation of re-occurrences of life-threatening cardiovascular events such as myocardial infarction (heart attack) and stroke for patients receiving larger dosages of aspirin and alteplase.^11,21^

The same magnitude of force reduction is not evident for tranexamic acid, indicating that it does not offer the same degree of adsorption and activity at the interface. While it is known that tranexamic acid behaves as an antifibrinolytic ‘clot promoter’ in the clinical environment, it also may act as a mild surfactant at the clot-liquid interface as indicated by the cohesive force-dosage relationship. However, there is uncertainty surrounding the effects of tranexamic acid on thromboembolic events and mortality, where in clinical trials in surgery, these remain inadequately assessed.^44^

Like other surface-active additives tested in this work, the compound is attracted to the clot-liquid interface in the experimental environment, despite the chemical environment in which it is located not being an accurate chemical representation of where it would be distributed in clinical applications—components of the blood clot may have lost biological activity. To our knowledge of the literature, data of this kind has not previously been reported for systems involving model blood clots. This outlines that some introduced surface-active chemistries to the bloodstream may interact with the solid–liquid interface by adsorbing to it from the bulk phase, and then provide resistance to thromboembolic cohesion *in vivo* upon particles colliding—similar adsorption behaviour may be also apparent for those species (e.g. proteins) that naturally reside in blood.

## Conclusions

This work is the first example of deployment of this technique in a field outside of oil and gas, and the first in a cardiovascular-focused application. The work from this manuscript provides direction to better inform the mechanisms of thromboemboli aggregation at instances of inter-particle collisions *in vivo*; a base experimental system was established with model blood clots and mechanically-relevant aspects of the measurement were explored. While in its infancy this simplistic experimental system does not accurately represent *in vivo* physiological conditions (e.g. chemically, thermally, fluid dynamically), this work is a necessary step towards understanding the governing properties of the measurement that enable statistical reproducibility and a baseline to probe the sensitivity of the measurement to the chemical environment. The measurement is time-dependent in the base system above the order of one minute; we hypothesize this to be due to a substantial increase in the contact area or a chemical reaction occurring at the point of contact. This may have significant implications for thromboemboli that collect in stagnant or recirculation zones in the arterial tree, whereby increased prospect of aggregation may take place. Below one-minute, less dependence was evident were the cohesion mechanism may be governed by a logarithmically distributed governing parameter such as contact area, where a surface roughness distribution may exist. The introduction of natural and pharmaceutical surfactant species in the continuous phase reduced the clotted blood particle separation force to within the detection limit of the technique at 1 wt%, where we hypothesize this implication to be due to surface-active material adsorbing to the solid–fluid interface. The technology may assist to inform the propensity for active-chemistries to adsorb to thromboembolic interfaces and reduce the aggregation potential *in vivo*. Future work will investigate how other physical parameters, which may be of critical importance, such as a distribution of surface roughness at the point of contact, affects the measurement. With some minor modifications to the experimental system, it is also possible to study particle adhesion to surfaces; vascular tissue, cardiovascular stents etc. Although the baseline measurement developed in this study may not represent the magnitude of the cohesive property *in vivo*, this measurement could be treated as a ‘worst-case scenario’ input within cardiovascular CFD simulations to predict thromboembolic trajectory and fate within the vasculature, given that naturally-present species (blood-derived proteins) reduce the measurement. Expanding on the momentum balance relationships developed by Mukherjee *et al*.^62^ by accounting for inter-particle contact force, this data provides additional context for examining how critical thromboembolic aggregation and deposition may be as intermediate phenomena in the development of an occlusion.^71^

## Supplementary Information

Below is the link to the electronic supplementary material.

Supplementary file1 (XLSX 22 kb)

Supplementary file2 (DOCX 510 kb)
