# Supplementary material for: Micromechanical Force Measurement of Clotted Blood Particle Cohesion: Understanding Thromboembolic Aggregation Mechanisms
Source: Cardiovasc Eng Technol. 2022 Apr 13;13(6):816–28. doi: 10.1007/s13239-022-00618-2 (PMC9750917; doi:10.1007/s13239-022-00618-2)
Supplement: Supplementary file 2 — Supplementary file2 (DOCX 510 kb) [file 13239_2022_618_MOESM2_ESM.docx]

1. SUpplementary MAterial
   1. **Assessable governing physio-chemical parameters of the baseline system**

There are a number of mechanical variables that govern the pull-off technique, previously described. Variables that can be studied with the current experimental arrangement are listed in Table 2, where a number of modifications can be made to probe the mechanics of the cohesive force measurement. Note: the temperature of the experimental cell may be controlled by the introduction of a temperature-controlled heating jacket. The influence of the majority of these parameters is discussed in the results section of this manuscript. With respect to the chemistry of the continuous phase that these particles are suspended in, there exists substantial opportunity for modifications to be made, particularly to study the effect of surface-active chemistries; cardiovascular pharmaceuticals, blood fractions etc.

**Table 2.** Experimental physio-chemical parameters and their modifications

| Parameter | Options | Comment |
| --- | --- | --- |
| Reproducibility | Number of ‘pull-off’ trials; independence between particle pairs; independence of blood products; sample age | 95% confidence boundary within 30% of average force |
| Particle Contact Location | (i) Unique in each pull-off vs. (ii) Identical in each pull-off | Prevent surface destruction |
| Pre-Load Force | (i) No force; (ii) Minimal contact force; (iii) High force | Ensure cohesive force is independent of pre-loading |
| Inter-particle Contact Time | (i) 1-10 seconds; (ii) 10-100 seconds; (iii) 100+ seconds | Contact time informs *mechanism* under study |
| Selection of Continuous Phase | (i) DI Water; (ii) Surface-Active Additives in DI Water; (iii) Whole blood in DI water; (iv) Blood serum; (v) Saline; (vi) Organic Phase | Maintaining chemical *simplicity* improves resolution when studying active chemistries |
| Temperature | (i) Room temperature; (ii) Body temperature | Note affect on vapour pressure |

*grey text indicates areas of potential future work

- 1. **Baseline System: distributed force properties**

The data presented in Figure 8 is a combination of all individual measurements in the base system, extracted from 31 data sets of 20 measurements each – this was the control study. These measurements were all conducted at low contact time (10 s) and low pre-load force. This collection of data for the base system is referred to as the ‘baseline’.


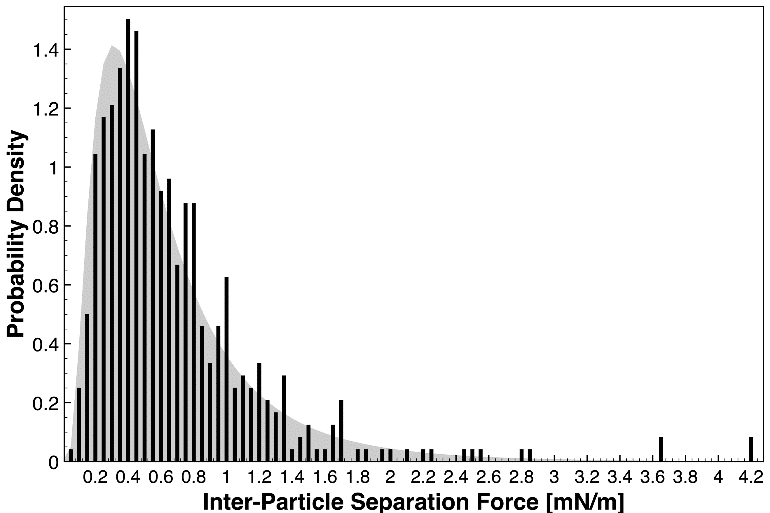


**Figure 8.** Distributed force properties with a lognormal distribution function overlaid. The black bars represent the probability density of the baseline measurement, where the bin range is 0.05 mN/m for individual measurements. The shaded grey area is utilised for visualisation of the distribution function.

Under baseline conditions, a culmination of 620 individual measurements from 31 data sets highlights the distributed properties which may inform the lognormality of cohesion mechanics evident between thromboemboli *in vivo*. The corresponding mean of the baseline measurements was 0.79 ± 0.06 [mN/m]. A lognormal distribution function was fit to the raw data, which is demarcated by a shaded grey area in Figure 8. Hypothesis testing was utilised to assess and visualise the lognormality in one or more variables involved in the measurement basis, and quantify an appropriate 95% confidence interval. In the current experimental arrangement, it is not possible to directly measure the inter-particle contact area. This may vary substantially between measurements, where a distribution of surface roughness may explain the lognormality of this data distribution – clotted blood particles visually exhibited asperities on the order of 10µm. Surface asperities and irregular characteristics below the detection resolution may have been present and influenced the measurement –reason to examine the baseline measurement across a statistically significant data set.

- 1. **Carbon fibre spring calibration: determination of spring constant, k**

Carbon fibre cantilevers used in this work were previously calibrated against a tungsten wire with known spring constant [81], in the work by Morrissy et al. [58]. The method described by Yang et al. [81] was used to determine the spring constant (0.025 ± 0.003 N.m^-1^) of a 24 mm tungsten wire. The calculated and measured spring constant (Figure 9) are within the overall uncertainty of the theoretical constant – where the pull-off distance and pull-off force is related linearly for deflections within 15% of the fibre’s length. Further details can be found in the work by Morrissy et al. [58].


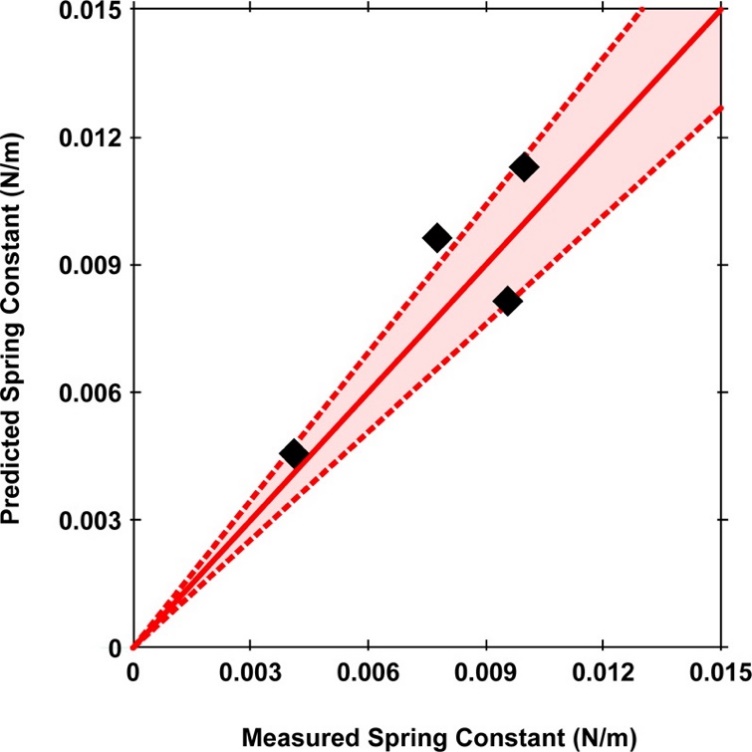


**Figure 9.** Comparison of the measured and predicted spring constant for four carbon fibres using the relative calibration method from Yang et al. [81]. The solid red line represents parity between measured and predicted spring constants, with the red shaded region representing the uncertainty bounds of the calculated spring constant. Reproduced with permission from Morrissy et al. [58]

- 1. **Reference Micromechanical Force studies: clathrate hydrate systems**

The following data is provided for reference, and is applicable to subsea oil & gas tiebacks – the foundational field application of the MMF technique. The system used to collect this data was composed of two cyclopentane clathrate hydrate particles, suspended in a continuous phase of cyclopentane. The inter-particle cohesive force of cyclopentane hydrate was measured as a function of additive concentration for four additives in Figure 10, respectively. The data highlights that the cohesive force decreases with increasing dosage concentration of hydrate-targeting surface-active additives – the vertical abscissa (Interfacial Tension [mN/m]) is synonymous with the dimensions of inter-particle cohesive force [mN/m]. In this study, the data shows that these surfactants may adsorb, as is hypothesized via a similar adsorption mechanism (noting that these surfactants are oil-soluble), to the hydrate-hydrocarbon interface at lower mass fraction than these species adsorb to the hydrocarbon-water interface, as indicated by the interfacial tension (IFT) data – an important outcome for the identification and development of hydrate-specific surfactants. The data used to collect the later information was an optical interfacial tensiometer. Further details can be found in Aman et al. [7].


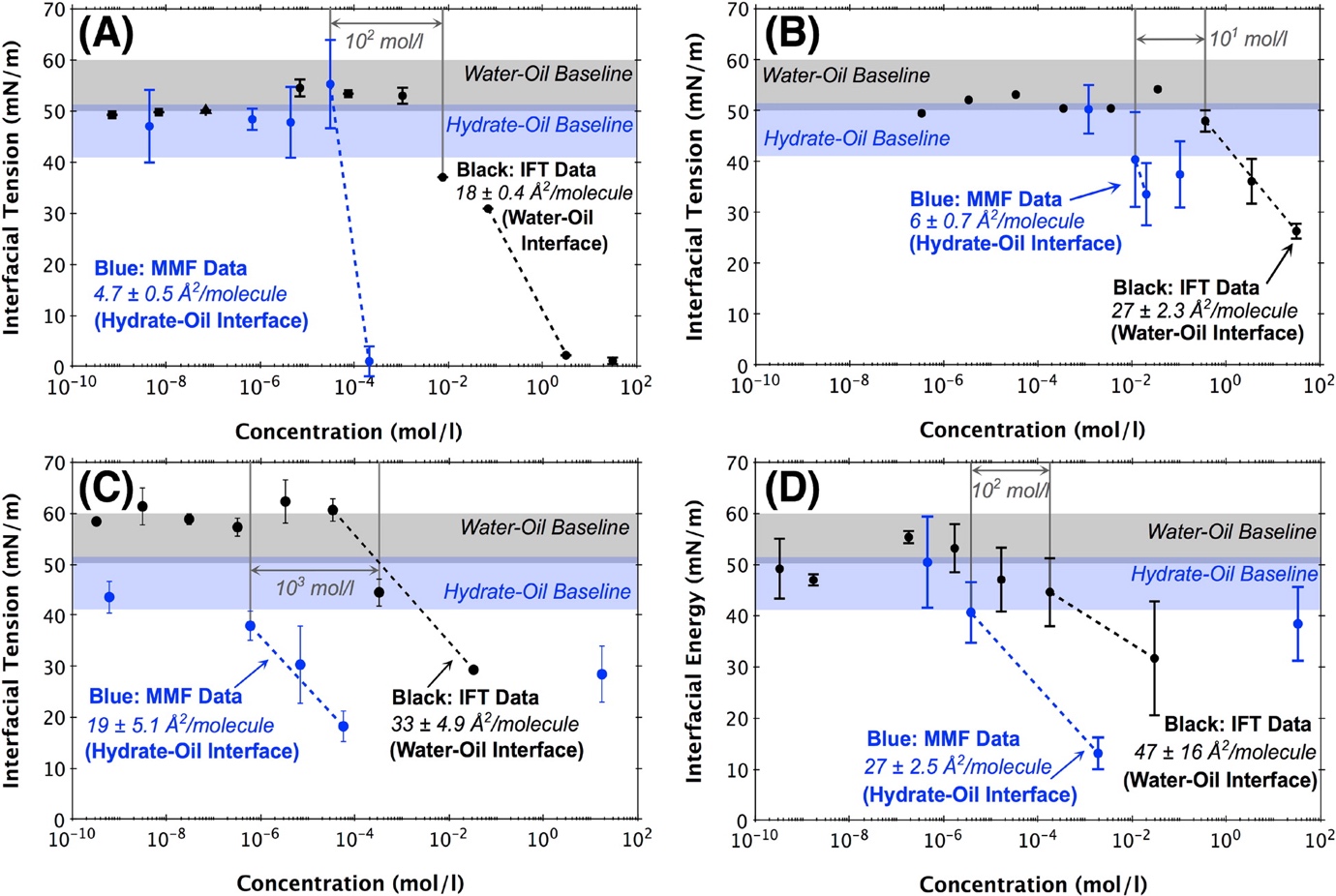


**Figure 10.** Water–oil (black) and hydrate–oil (blue) interfacial tension as a function of additive concentration: (A) dodecyl benzene sulfonic acid, (B) arachidic acid, (C) pyreneacetic acid, and (D) triphenylacetic acid. Error bounds represent 95% confidence intervals, and dashed lines are provided to identify estimated adsorption isotherms. Each data point is based on 40–120 individual pull-off trials, described in the experimental procedure. Reproduced with permission from Aman et al. [7].
